# Assessment of emotional predisposition in dogs using PANAS (Positive and Negative Activation Scale) and associated relationships in a sample of dogs from Brazil

**DOI:** 10.1038/s41598-019-54645-6

**Published:** 2019-12-05

**Authors:** Carine Savalli, Natalia Albuquerque, Angélica S. Vasconcellos, Daniela Ramos, Fernanda T. de Mello, Daniel S. Mills

**Affiliations:** 1 Federal University of São Paulo, Santos, Brazil; 2 University of São Paulo, São Paulo, Brazil; 3 Pontifical Catholic University of Minas Gerais, Belo Horizonte, Brazil; 4 PsicoVet, São Paulo, Brazil; 5 Paulista University, São Paulo, Brazil; 6 Animal Behaviour Cognition and Welfare Group, School of Life Sciences, University of Lincoln, Lincoln, UK

**Keywords:** Ecology, Behavioural ecology

## Abstract

The English version of the Positive and Negative Activation Scale (PANAS) is a useful tool for the assessment of dog temperament, helping to identify highly sensitive individuals that could be at risk of developing fears, phobias and anxiety problems, and potentially depressive states. This study evaluated the association between dogs’ and owners’ characteristics and dog temperament in Brazil. To accomplish this, we adapted and validated a Portuguese language version of PANAS for dogs. Data from 1744 owner-dog dyads were analysed and a two-factor structure similar to the original PANAS was revealed that met the requirements for validity and internal consistency. We found that dogs owned by women, neutered dogs and those who live in single-dog households show higher negative activation. Moreover, the older the owner, the less the negative activation for dogs that are bought. We also found that the older the dog, the less the positive activation, but this score is higher in dogs that sleep inside the house. Interestingly, mixed-breed dogs scored higher for both negative and positive emotional activation compared to purebreds. These findings alongside the particular profile of dogs in Brazil, including its large population of mixed-breed, emphasise the value of cross-cultural investigations in order to develop a full understanding of dog behaviour.

## Introduction

Interindividual behavioural differences may be linked, among other aspects, to demographic and morphological features^1^ and have fundamental fitness consequences in terms of access to resources and decision-making, especially in the social sphere^2^. Temperament can be associated with several factors; it has a genetic component, it can be observed in an individual from an early age^3^ and it can affect dogs’ abilities to deal with social and environmental challenges^4^. Several studies have investigated differences in temperament in dogs^5^. Gartner^5^ in his review proposes an interesting model for studying the relationship between intrinsic and extrinsic factors affecting temperament in the anthropogenic environment since dogs have to interact with and adapt to human beings daily. Human-dog association provides a special scenario for studying how a complex interspecific social environment combined with demographic factors can influence animal behaviour. The environment where an animal lives plays an important role in shaping its temperament. Animals behave according to their local conditions, and the ability to appropriately respond to one’s current environment is adaptive, since natural selection favours animals whose behaviour increases fitness^6^. Indeed, behavioural variation is maintained by changes in selective pressures due to differences in intrinsic and extrinsic conditions^6^ and the latter might also reflect local cultural differences^7–10^.

Temperament can be assessed and measured in a variety of systematic ways. A common schema uses bivariate categories associated with core affect, such as positive and negative activation^11^, although temperament can also be defined in relation to other biological parameters to indicate how an individual tends to behave in a diversity of more specific conditions. This operational approach, which may also be considered a behavioural profile rather than temperament, is useful especially when making comparisons across animals and populations facing the same context, such as being in unfamiliar environments or exposed to a disturbing stimulus. There are two common methods used to achieve these goals: the direct observation of the behaviour, and the use of questionnaires and scales for psychometric evaluation (as frequently used to assess human personality). In the case of domestic dogs, owners spend a lot of time with them and, given their knowledge about the daily behaviour of their dogs, they can potentially provide a major contribution to the investigation of their emotional functioning and temperament^12,13^. Thus, in the psychometric approach, owners can answer questions regarding their dogs’ response tendencies in relation to different stimuli and events^14^. The questionnaire-based approach has been shown to have the potential to be reliable and advantageous, especially because it allows efficient data gathering from large sample sizes, and data collection from subjects over a great number of situations, by respondents from different geographic regions^15^. If validated, questionnaires can be made available to stakeholders such as veterinarians, trainers and other professionals to be used in their daily practice. However, it is also important to appreciate the potential cultural sensitivity of questionnaires. Not only may different cultures impose different environments on a subject which may shape its temperament (and associated norms), but the items used may be of different value and be interpreted differently. These important factors appear to have been largely overlooked in the companion animal temperament literature.

The Positive and Negative Activation Scale (PANAS), for example, first elaborated for humans^16^ and more recently adapted for dogs^17^, has been found to be a useful tool for the assessment of dog temperament traits in the English-speaking world. This questionnaire was developed to assess responsiveness to rewarding and aversive experiences, namely positive and negative activation domains, and aids in the identification of animals at risk, e.g. high negative activation is associated with an increased risk of developing fears, phobias and anxiety problems. Moreover, differences in emotional sensitivity can guide the diagnosis of behavioural problems and treatment recommendations^18^ and predict different responses to behaviour-modification programs or working success^19^. The approach proposed by Sheppard and Mills^17^ emphasises the identification of behavioural traits with psychobiological foundations considering the complex and nuanced world where dogs live. The focus on positive and negative activation generates a measure that allows predicting behavioural tendencies across diverse contexts, in different reinforcing situations. The original validated version of PANAS for dogs in English has 21 questions aiming to access two broad behavioural domains related to reinforcement sensitivity^20^. Each question consists of a statement (e.g. “*your dog is easily excited*”) and provides a five-point Likert scale (from strongly agree to strongly disagree) for the respondent, in addition to the option “Not applicable”. Positive and negative scores of PANAS are not opposites but related orthogonally. Higher negative scores can indicate emotional disturbance or distress, startle responses to unpredictable stimuli and low tendency to habituation, while lower negative scores can indicate calmness. On the other hand, higher positive scores can indicate active and enthusiastic engagement with the environment and lower positive scores could indicate apathy or depression^17^. Recently, some of the PANAS items were used by Braem and colleagues^21^ to develop a *highly sensitive dog questionnaire*, which aimed at identifying “canine sensory processing sensitivity” and higher vulnerability to stress. The instrument was also used recently to analyse the interaction between the health condition of pet dogs and their positive and negative affect. The results demonstrated that lower positive scores were associated with pain experience^22^, which reinforces its usefulness in the veterinary setting.

Studies that investigate the association between dogs’ temperament and demographic features using the approach of positive and negative emotional activation, as provided by PANAS, are missing in the scientific literature. However, some studies using other instruments have evaluated relevant aspects of behaviour. Calmness, for example, was found to be associated with the dog’s age and reproductive status^8^. Older dogs are calmer; on the other hand, neutered dogs were reportedly less calm than intact dogs^8^. Owners that interact more and have previous experience with ownership often have calmer dogs^23,24^. Increased calmness is also associated with greater time spent with the owner^8^. For female dogs, more people around was related to higher calmness, however, no such effect was found in male dogs. Boldness was also associated with the sex, age and reproductive status of dogs^8,10^. Boldness seems to decrease with age, male dogs being bolder, as are intact dogs. Moreover, the dogs of women were reported to be less bold^8^. The owner’s gender has been associated with variation in the owner-dog relationship style and the way owners interact with their dogs^25,26^. Kotrschal and colleagues^9^ and Evans-Wilday and colleagues^27^ have also found differences in the way women and men communicate with their dogs, which may influence dogs’ behaviour. Although breed differences in reaction to novelty are described (see Mehrkam and Wynne^28^ for a review), the extent to which these are genetically determined is questionable, since variation within breeds may be higher than between breeds^29^ and experience with the surroundings may be critically associated with increased fear and excitability^30^. Thus, the extent to which the way dogs respond to emotionally charged situations is associated with their demographic characteristics must be better understood, with attention given to the potential importance of cultural differences, e.g. differences in dog management style.

According to Wallis and colleagues^31^, in 2016 a quarter of all households in the UK, 33% in Hungary, and 44% in the USA owned a dog. Brazil not only has the second highest dog population in the world, but in 2013 it was found that 44.3% of households have at least one dog^32^. Surprisingly, in Brazil the study of canine behaviour is a recent field of scientific interest, and studies on the relationship between owners and their dogs are rare. Data from a recent behavioural caseload^33^ has pointed to Brazilian cultural specificities: e.g. lifestyles in many cities are perhaps more frustrating and restrictive in a range of ways, and there is an increased risk of noise fear and phobias in pet dogs, which may be linked to greater use of fireworks throughout the year in this country.

Therefore, the aim of this study was first to assess the concurrent validity of a Portuguese version of PANAS compared to the original English version and to investigate how intrinsic and extrinsic characteristics of dogs influence their temperament in Brazil. The characteristics considered were: dogs’ sex, age, breed and reproductive status, as well as owner demographic features and family structure, walking habits, the place where the dog sleeps and the presence of other dogs in the house.

## Methods

### Ethics statement

This research was approved by the ethics committee of the Federal University of São Paulo and complies with Brazilian legislation. We obtained the informed consent from all respondents before they answered the online questionnaire.

### Translation and cultural adaptation of the PANAS questionnaire

Following the method proposed by Beaton and colleagues^34^, this phase included the translation, back-translation, semantic and cultural adaptation and testing of the adapted questionnaire. Two versions in the Portuguese language were produced by two independent researchers, then these versions were compared and combined to obtain a single version. Subsequently, this Portuguese version was independently back-translated and compared to the original English version in order to confirm whether the Portuguese questions were precisely reproducing the original English questions. After some adjustments, a Portuguese pilot version was tested in a small sample (N = 40) in order to evaluate whether Brazilian participants understood the questions’ wording and meaning. Finally, adjustments were discussed by all native Brazilian authors of this manuscript and a final version was agreed upon by consensus (Supplementary Information).

A link to the final version was embedded in the website of the Centre of Research of Well-being and Human Behaviour-São Paulo. Participants were recruited via social media (i.e. advertisements on Facebook, Twitter, etc.), emails were directly sent to potential respondents and advertisement posters placed at strategic places, such as universities, veterinary clinics, pet shops and animal-related conferences. The survey remained active online from mid-November 2017 to the end of April 2018. Each owner could answer the questionnaire for one dog only so as to avoid dependence within the dataset and pseudo-replication.

### Participants and missing pattern of PANAS questions

We obtained 2054 complete responses from owners representing all regions of the country. Participants younger than 18 years old were excluded in accordance with our ethical approval. For 89 participants (4.9% of the whole sample), more than 20% of PANAS items were answered as “not applicable”. These questionnaires were also excluded from the data set, resulting in a final sample of 1744 analysable responses.

The mechanism behind missingness (i.e. “not applicable” responses) was investigated. For each item of the PANAS, a variable indicating which responses were missing or not was created, and the correlations between these indicators were then analysed. These indicators were weakly or not correlated at all (90% of Spearman correlations between all pairs of indicators were under 0.30), which means that the assumption of random occurrence of missing data is reasonable. In addition, the item *“Your dog tries to escape from the garden”* was removed from subsequent analyses because it had a high missing rate (14% of all respondents chose “not applicable” for this question), which can indicate that a large proportion of Brazilian owners may not live in houses with gardens or backyards, hence this item was excluded. Only a small proportion of the other 20 questions were missing (2%) and they were treated with statistical imputation to complete the records.

### Statistical analysis

#### PANAS adaptation and validation

First, the distribution of the 20 PANAS items was analysed. Descriptive statistics were calculated to identify strong deviations from a symmetric distribution. Items with high rates of responses in the extremes of the scale (median = 5.0 and mean >4.0 or median = 1.0 and mean <2.0) were excluded from the following analyses since these indicate poor differentiation among subjects.

After excluding items with strong asymmetry, a cross-validation analysis was applied. Data from 1744 participants were randomly divided into two subsamples with 872 dogs each. The first sample was submitted to an exploratory factor analysis (EFA). The factor structure obtained from the first subsample was tested in the second subsample through a confirmatory factor analysis (CFA). For the EFA, the extraction method used was the principal factors with a varimax rotation. The adequacy for using the EFA was evaluated through the Bartlett’s sphericity test and the Kaiser-Meyer-Olkin (KMO) measure of sampling adequacy. The Kaiser’s eigenvalue rule was considered to define the number of factors. Subsequently, items were retained if their loading on one of the factors were greater than 0.35 and relatively lower on others (i.e. the lower difference in loading between the two factors was 0.24). Items that did not fulfil this criterion were excluded. Internal consistency of the extracted factors was evaluated using the Cronbach’s alpha coefficient. For the CFA, the Root Mean Square Approximation of Error (RMSEA) and Comparative Fit Index (CFI) were preferred as goodness-of-fit criteria, since the Chi-Square test is excessively sensitive to a large sample size.

#### Influence of intrinsic and extrinsic characteristics on dog’s temperament

Based on the final factor structure and considering the whole valid data set, negative and positive activation scores were calculated. To calculate the score for each construct, items were scored as per Sheppard and Mills (2002) and then divided by five times the number of items making up the construct, to give a value between 0.2–1. Scores for each construct were then standardised, and a multiple regression analysis was applied to investigate whether these temperament dimensions were associated with dogs’ and their owners’ demographic characteristics, and their relationship. A significance level of 1% was adopted for selecting strong associations only. Characteristics tested were: (i) gender, age and educational level of the owners; (ii) sex, age, breed (mixed-breed or purebred) and reproductive status (intact or neutered) of dogs; and (iii) variables concerning the relationship: whether or not owners take their dogs for walks, the place where dogs sleep (inside or outside the house), the presence of other dogs in the house (single or multi-dog households), the way dogs were acquired (adopted, bought, received as gift or other origin), presence of other people in the house (i.e. whether or not owners live with a partner/family), and whether or not the dog was the first one to be owned by the respondent. A model with main effects and all first-order interactions was fitted to examine the relevance of the interactions. In a second step main effects and only significant interactions were submitted to the selection procedure *stepwise*. In the current study the term “mixed-breed” does not refer to a mixture of known breeds, but rather to an uncertain ancestry.

## Results

### PANAS adaptation and validation

Descriptive measures of each item for the 1744 responses are presented in Table 1 (the number of the items presented in this table will be used throughout the text). According to the criteria described in the methods, items 2, 12 and 17 were removed from these analyses due to strong asymmetry, i.e. a large proportion of responses in the extreme of the scale, which, consequently, resulted in poor differentiation among dogs.

**Table 1:** Descriptive measures for the items of PANAS. Items were presented and numbered (values in bold indicate high asymmetry in scores).

Items*	median	mean	Standard deviation
1-Your dog is rarely frightened	2.000	2.550	1.289
2-Your dog becomes very excited when it is about to go for a walk (e.g. when it sees its lead, or when it hears “walkies”, etc.)	**5.000**	**4.241**	1.157
3-Your dog is easily startled by noises and/or movements	3.000	2.892	1.368
4-Your dog is very persistent in its efforts to get you to play	4.000	3.463	1.313
5-Your dog shows little interest in its surroundings	4.000	4.030	1.107
6-Your dog appears nervous and/or jumpy for several minutes after it has been startled	2.000	2.170	1.278
7-Your dog is easily excited	4.000	3.539	1.251
8-Your dog has a specific fear or phobia	2.863	2.863	1.627
10-Your dog appears calm in noisy, crowded places	3.000	2.880	1.408
11-Your dog is full of energy	4.000	4.019	1.201
12-Your dog is frightened by noises from the television or radio	**1.000**	**1.440**	0.944
13-Your dog usually appears relaxed	2.000	2.035	1.149
14-Your dog is lazy	3.000	3.294	1.451
15-Your dog adapts quickly to changes in its environment (e.g. being cared for by different people, moving house or a family member leaving home)	2.000	2.543	1.319
16-Your dog appears afraid of the vacuum cleaner or any other familiar household appliance	2.664	2.664	1.499
17-Your dog requires a great deal of encouragement to take part in energetic activities	**5.000**	**4.133**	1.203
18-Your dog persists in being naughty despite being told off for the behaviour	3.000	2.765	1.460
19-Your dog appears calm in unfamiliar environments	2.660	2.660	1.337
20-Your dog is very boisterous	2.000	2.643	1.381
21-Your dog appears unsettled by changes to its routine (e.g. if it is not fed at the usual time, if it is left alone for longer than usual)	2.000	2.604	1.369

*(The item numbered as 9 is not presented in this table because it was previously excluded due to high frequency of missing responses).

The remaining 17 items were submitted to an EFA considering the first subsample of 872 dogs. The Kaiser’s eigenvalue rule suggested the extraction of four factors; the first and second factors accounted for 21.5% and 15.1% of the total variability in the correlation matrix, respectively. Altogether these two factors accounted for a greater proportion of the variation (36.6%) while remaining factors accounted for less than 8%. Therefore, the solution with two factors was considered appropriate. We proceeded by evaluating the loading of each item on these two factors. Item 13 (“*Your dog usually appears relaxed*”) loaded 0.38 on the first factor and 0.33 on the second factor, therefore it was removed since it was ambiguous in the Brazilian sample.

For the final solution, the Bartlett’s sphericity test (p < 0.001) indicated that the correlation matrix was not an identity matrix and the KMO = 0.801 indicated that it was adequate to group items into a set of two interpretable factors. The items that loaded higher on the first factor were 1, 3, 6, 8, 10, 15, 16, 19, 21, accounting for 21.7% of total variation, and the items that loaded higher on the second factor were 4, 5, 7, 11, 14, 18, 20, accounting for 16.0% of total variation (Table 2). Therefore, the final factor structure yielded 16 items grouped into the two factors that accounted for 37.7% of the common variance. The first factor included items interpreted as negative activation and the second factor included items interpreted as positive activation in the same way as the original PANAS in English. The first and second factors presented good internal consistency: Cronbach’s alpha coefficients were 0.77 and 0.71 respectively, i.e. both grouping of items emphasise a single idea or construct.

**Table 2:** Rotated factor loadings of the first and second factors obtained in the EFA with Varimax rotation, the proportion of variance explained for each factor and the reliability coefficient Cronbach’s alpha for each factor.

Items	Factor		Original PANAS
	1	2	
1-Your dog is rarely frightened	**0.668**	−0.039	Negative activation
3-Your dog is easily startled by noises and/or movements	**0.738**	0.013	Negative activation
6-Your dog appears nervous and/or jumpy for several minutes after it has been startled	**0.630**	−0.007	Negative activation
8-Your dog has a specific fear or phobia	**0.595**	−0.098	Negative activation
10-Your dog appears calm in noisy, crowded places	**0.663**	0.096	Negative activation
15-Your dog adapts quickly to changes in its environment (e.g. being cared for by different people, moving house or a family member leaving home)	**0.501**	0.004	Negative activation
16-Your dog appears afraid of the vacuum cleaner or any other familiar household appliance	**0.530**	0.005	Negative activation
19-Your dog appears calm in unfamiliar environments	**0.552**	0.120	Negative activation
21-Your dog appears unsettled by changes to its routine (e.g. if it is not fed at the usual time, if it is left alone for longer than usual)	**0.465**	0.222	Negative activation
4-Your dog is very persistent in its efforts to get you to play	0.097	**0.655**	Positive activation
5-Your dog shows little interest in its surroundings	−0.069	**0.408**	Positive activation
7-Your dog is easily excited	0.234	**0.630**	Positive activation
11-Your dog is full of energy	−0.069	**0.788**	Positive activation
14-Your dog is lazy	−0.103	**0.518**	Positive activation
18-Your dog persists in being naughty despite being told off for the behaviour	0.120	**0.456**	Positive activation
20-Your dog is very boisterous	0.063	**0.721**	Positive activation
Variance	3.48	2.55	
% Variance	21.7%	16.0%	
Cronbach’s alpha	0.77	0.71	

Finally, the two-factor structure for 16 items was tested in the second subsample of 872 dogs through a CFA. Recommended indices to evaluate goodness of fit in CFA confirmed that the hypothesised model is, in fact, appropriate (RMSEA = 0.058; CFI = 0.901). In the original PANAS, three facets of the positive activation items are described; however, since items were removed from the Brazilian version, and some of the subfactors were made up of only two items, this substructure was not explored further.

Considering the final two-factor structure presented in Table 2, standardised scale scores for positive and negative activation were calculated to investigate the influence of the dogs’ social and demographic characteristics on these temperament dimensions.

### Sample profile

The sample consisted of dogs with an average age of 65.1 months (standard deviation = 44.9), both sexes were well represented (females: 51.4%, males: 48.6%) and 60.6% of them were spayed/neutered (68.2% of female dogs and 52.4% of male dogs). The sample comprised mixed-breed (35.4%) and purebred dogs (64.6%) with more than 60 breeds represented: the most frequent were shih tzu (6.0%), poodle (4.3%) and golden retriever (4.0%). Thirty-seven percent were adopted, 36.9% were bought and the remaining came to the owners by other ways. Almost half of them (45.8%) were kept in multi-dog households and 72.5% of dogs slept inside the house. A considerable proportion of respondents (21%) never walked their dogs.

Among the 1744 owners, 77.8% were women and the age ranged from 18 to 77 years old (mean = 35.3, standard deviation = 11.7). Participants were mostly well educated, 66.9% had obtained at least a college degree. Eighty-seven percent lived with a partner or family and 81.4% of the respondents had previously owned another dog. The two most represented Brazilian regions were the Southeast (75.9%) and the South (9.1%).

### Relationship between dogs’ and owners’ characteristics and dogs’ temperament

For all 1744 dogs the average of negative scores were 0.53 (standard deviation = 0.16) and the average of positive scores were 0.68 (standard deviation = 0.16). The results of the two multiple linear regression analyses after the *stepwise* selection of variables that were significantly associated with dogs’ temperament measured by standardised PANAS scores can be seen in Tables 3 and 4.

**Table 3:** Estimated parameters and statistics of the final model selected for the NEGATIVE activation score. (df: degrees of freedom).

Variable	df	Parameter estimate	Standard Error	t Value	p	Direction of NEGATIVE score
Intercept	1	−0.27130	0.07146	−3.80	0.0002	
Owner gender	1	0.32674	0.05742	5.69	<0.0001	Owner female > Owner male
Dog Breed	1	0.16300	0.06835	2.38	0.0172	Mixed-breed > Purebred
Reproductive status	1	0.13758	0.05017	2.74	0.0062	Spayed/neutered > Intact
Presence of other dogs	1	−0.19360	0.04659	−4.16	<0.0001	Multi-dog < Single dog
Owner age	1	−0.00125	0.00351	−0.36	0.7212	
Source (Adopted)	1	0.04688	0.06991	0.67	0.5026	
Source (Bought)	1	−0.13285	0.06173	−2.15	0.0315	Other origin > Bought
Owner age*Source (Adopted)	1	−0.00270	0.00493	−0.55	0.5847	
Owner age*Source (Bought)	1	−0.01355	0.00482	−2.81	0.0050	The older the owner, the lower the negative score for dogs that were bought

**Table 4:** Estimated parameters and statistics of the final model selected for the POSITIVE activation score. (df: degrees of freedom).

Variable	df	Parameter estimate	Standard Error	t Value	p	Direction of POSITIVE score
Intercept	1	0.78390	0.05347	14.66	<0.0001	
Age of dogs	1	−0.00876	0.00048209	−18.17	<0.0001	The older the dog, the lower the positive score
Place where dog sleep	1	−0.35196	0.04855	−7.25	<0.0001	Inside > outside
Breed	1	0.11961	0.04532	2.64	0.0084	Mixed-breed > Purebred

Regarding the negative activation scores, the model considering the main effects and all first-order interactions indicated that the interaction between the owner’s age and the way the dog was acquired (adopted, bought or other origin) should be considered. All other first order interactions were not significant in this model (p > 0.01), therefore, they were not taken into account. A *stepwise* selection procedure in the model with all main effects and this significant interaction led to the final model presented in Table 3. Once the interaction between the owner’s age and the way the dog was acquired was selected in the final model, the main effects linked to this interaction were also included only for the purpose of estimation. By including these two main effects in the final model, the dogs’ breed significance became slightly greater than the significance threshold of 1% (Table 3), however, since it had been initially selected, it was retained in the final model. According to the final model, owner’s gender, dog’s breed and reproductive status, presence of other dogs in the house and the interaction between owners’ age and the way dogs were acquired were associated with negative emotional activation. Dogs from female owners had greater negative activation scores than dogs from male owners. Mixed-breed dogs showed more negative activation than purebred dogs. Dogs that were spayed/neutered had higher negative activation than intact dogs. Dogs that lived alone (i.e. with no other dogs) showed greater negative activation than dogs living in multi-dog households. The interaction between owners’ age and the way dogs were acquired indicated that the older the owner, the lower the negative activation score for dogs that were bought. This association was not observed for other sources of acquisition.

Regarding the positive activation scores, the model considering the main effects and all first-order interactions indicated that the interactions between the owner’s educational level and sex of the dog, between reproductive status and sex of the dog and between owner’s age and dog’s age should be considered. All other first-order interactions were not significant in this model (p > 0.01). After the *stepwise* selection procedure in the model with all main effects and these three interactions, only three main factors appeared relevant to explain positive emotional activation in dogs and were included in the final model (estimated parameters and statistics are presented in Table 4): dogs’ age, breed and the place where the dogs sleep (inside or outside the house); the older the dog, the lower the positive activation score, mixed-breed dogs showed greater positive activation scores than purebred dogs, and dogs who sleep inside the house showed more positive activation.

## Discussion

The translated and adapted version of PANAS for Brazilian language and culture has concurrent validity with the two behavioural dimensions corresponding to the positive and negative dimensions of the original English-version of the questionnaire^17^. We also found that the negative activation score in the Brazilian sample was associated with the owners’ gender, dogs’ breed and reproductive status, the presence of other dogs in the house and the owners’ age when dogs were bought. Positive activation showed fewer relationships but pointed to an effect of the dogs’ age and breed and the place where they sleep in the house (inside or outside). The validated Brazilian PANAS is a product of this study, which provides Brazilian owners and professionals who rely on animal behaviour data (e.g., veterinarians, psychologists, ethologists) a specific tool to help identify dogs at risk of specific problems and relapse following treatment due to their temperament, for example those with fears, phobias and anxiety problems; the tool also potentially facilitates a greater ability to predict a dog’s personal and/or work capacity.

The two-factor structure is similar to the original tool in English, but it is important to note that the tool did require cultural adaptation. We believe this is the first time a psychometric instrument for non-human animals has been adapted for validation in such a way, rather than simply being translated for use in another country. Nonetheless the broad structure was comparable. The first factor extracted included items that were interpreted as negative activation, related to fearful or anxious states, reactions to changing environments and startle responses. Fear and anxiety were characterised by mood descriptors such as *afraid*, *fear*, *nervous* and *calm* (in the sense of lack of calmness). The second factor extracted included items that were interpreted as positive activation, related to energy, interest, persistence and excitement as a single factor. It was characterised by mood descriptors such as *interest*, *excited*, *energy* and *lazy* (in the sense of lack of laziness). The cross-validation strategy strengthened the results, and measures of internal consistency suggested that positive and negative activation scales were each represented as separate constructs. The two scales for the 16 items accounted for 37.7% of the common variance within the Brazilian sample, close to that obtained with the 21 items in the original English version (43.1%), being, therefore, a valid tool to use in Portuguese language.

In the current study, we hypothesised that the dogs’ emotional activation would be related to some of the demographic characteristics of dogs, owners and their relationship. To investigate these connections, we used regression models with a conservative level of significance to detect only strong associations, since in large samples small effects can be mistakenly considered important. Dogs owned by women had higher negative activation ratings than dogs owned by men. This difference may have arisen due to distinct gender-dependent perception, rating or owning styles. In an attachment test with direct observation of people interacting with their dogs, Prato-Previde and colleagues^35^ did not find gender differences in affiliative and play behaviours, however they did find differences in verbal communication: women started talking earlier once in contact with their dogs and talked more than men. Evans-Wilday and colleagues^27^ found a similar tendency among women to self-disclose much more to their dogs than men, and differences between men and women in their social behaviour^36^, coping strategies^37^ as well as in their interactions with, and attitudes towards animals^26^ have also been reported. Women report more fear and less happiness than men after a social stress test^36^, have lower cortisol variability (an index used as a measure of health status and well-being^37^), and show stronger emotional bonds with companion animals than men show^9,27,35^. It is possible that these differences affect dogs’ temperament or the perception of this.

We also found that mixed-breed dogs had higher negative activation ratings than purebred dogs. Given the huge diversity of purebreds found in the studied sample, it was not possible to analyse specific breeds, but it is worth noting that it was this factor and not adoption from a rescue centre that was predictive, indicating that it is not the rescue experience that impacts most on the dogs’ temperament in our study. Bamberger and Houpt^38^ and Blackwell and colleagues^39^ found that crossbreeds were at higher risk for behavioural problems. One might suggest that if negative affective states are at the root of some (if not most) of the common canine behaviour problems seen by specialists^40^, this could explain, at least in part, our results. Many mixed-breed dogs in Brazil have been strays (i.e. lived on the street) and are frequently victims of mistreatment and abuse, which may engender higher negative emotional activation, for self-preservation or as a consequence of their experience. Interestingly, in the current study, mixed-breed dogs also had higher positive emotional activation, which may be linked to an opportunistic life-style prior to living with a family, i.e. higher activity levels and ability to take resources when on the street. This further highlights the independence of positive and negative activation as measures of core affect.

Dogs’ reproductive status was also related to negative activation, with neutered dogs showing higher negative activation than intact ones. This finding is consistent with those of Braem and colleagues^21^ and Sherman and Mills^41^. The former found that spayed/neutered dogs scored higher than intact ones in a scale that assesses the canine sensory processing sensitivity, but this effect was marginal and only true for male individuals, while the latter reports that neutered females are at greater risk of noise fears. The impact of castration in dogs’ behaviour is still not clear. Farhoody and colleagues (2018), for example, found no association between gonadectomy and problem behaviours associated with aggression^42^. Our results add to the growing body of evidence that highlights the complex relationship between neutering and problem behaviours^42–46^, which requires further investigations.

We also found higher negative activation scores in dogs from single-dog households in comparison with multi-dog households. This result may reflect the widely believed potential benefits played by dog-dog interactions, which will be more likely in multi-dog households. For instance, in a study on noise fear, dogs who lived without other dogs had more changes in their salivary cortisol after a noise exposure^47^. It may be the case that living with other dogs, which provides more opportunities for social development, enables social buffering and the emergence of coping strategies and reduces their sensitivity to negative events and stimuli^48^. Alternatively, (or in addition) it could be argued that multi-dog owners may be more experienced in rearing dogs and/or training them, and thus deal better with day-to-day challenges and more sensitive dogs, which could then alleviate their dogs’ negative affective predisposition.

Interestingly, the estimated equation showed that negative activation is inversely related to the owner’s age, only for dogs that were bought. Although this study did not present a within-subject design, the association observed suggests that, for dogs that were bought, the older the owner the less sensitive the dog is to negative stimuli. Previous studies have suggested that the source of acquisition of the dogs can influence their behaviour, with dogs bought from pet shops showing more fearful behaviours^39,49^, which could be related to the restricted and stressful environments in which they have developed. Older owners may also have more experience in raising their dogs, however, this hypothesis should be better explored in further studies.

Regarding positive activation, it is not a surprise that the older the dog, the less the positive activation score. In fact, according to Wallis and colleagues^31^ the older the dogs, the fewer daily activities people in general offer them, which consequently decreases their vitality. These authors also pointed out that older dogs have more sensory and health problems, which usually compromise their engagement in activities such as playing or physical exercises; indeed, depressive states reflect lowered positive activation^50^. Our results are in accordance with these findings.

Finally, dogs who sleep inside the house scored higher for positive activation. These dogs may spend more time in direct contact with their owners, who may play with them more but also inadvertently reinforce a range of attention-seeking behaviours^51^, which may be represented through excitability and positive activation, at least from the owners’ point of view. However, by contrast, Kubinyi and colleagues^8^, working with Hungarian owners, found that calmness increased with a greater time spent together. Although we did not analyse the specific place where dogs sleep inside the house, it is common for owners to share their beds with dogs. It is already known that the co-sleeping may have positively and negatively impact on human sleep quality^52,53^, nevertheless the relationship between where dogs sleep and emotional activation in them needs further investigation, especially in relation to potential cultural differences.

## Conclusion

This study has highlighted the need for cultural sensitivity, not only in relation to appreciating the range of factors that might shape a dog’s temperament but also in the design and translation of instruments aimed at assessing this aspect of personality. Owners who are reading, interpreting, and responding to the items about their dogs are embedded within a culture. Characteristics such as communication, attitudes to animals and interaction styles can be influenced by the culture, which makes the adaptation and validation of the instrument extremely important. Our adapted Brazilian PANAS questionnaire met the requirements of validity and internal consistency which assures that it is a robust and useful tool, and so the conclusions in relation to the demographic factors affecting a dogs’ temperament are robust. The associations found between dogs’ and owners’ characteristics and their interactions, with both positive and negative emotional activation have many potential explanations and future studies should not only seek to establish whether such associations are consistent across cultures, but also aim to elucidate the mechanisms underpinning the findings reported and any differences that might relate to cultural factors.

## Supplementary information

PANAS Portuguese
