# Supplementary material for: Assessment of emotional predisposition in dogs using PANAS (Positive and Negative Activation Scale) and associated relationships in a sample of dogs from Brazil
Source: Sci Rep. 2019 Dec 5;9:18386. doi: 10.1038/s41598-019-54645-6 (PMC6895085; doi:10.1038/s41598-019-54645-6)
Supplement: Supplementary file 1 — PANAS Portuguese [file 41598_2019_54645_MOESM1_ESM.doc]

## Title: Assessment of emotional predisposition in dogs using PANAS (Positive and Negative Activation Scale) and associated relationships in a sample of dogs from Brazil

## Authors: Carine Savalli, Natalia Albuquerque, Angélica S. Vasconcellos, Daniela Ramos, Fernanda T. de Mello, Daniel S. Mills

## Supplementary Information: Portuguese adapted and validated version of PANAS (Positive and Negative Activation Scale) for dogs

##

## PANAS - Escala de ativação positiva e negativa para cães

Para cada uma das afirmativas abaixo, por favor, marque um “X” na opção que mais precisamente descreve seu nível de concordância quanto a como seu cão, em geral, se comporta naquela situação. Por favor, considere em que medida o comportamento do seu cão é de intensidade similar e ocorre tão frequentemente quanto descrito. Por exemplo, se no item 2 seu cão **sempre** fica **um pouco** excitado quando está prestes a sair para passear, você concordaria razoavelmente com a afirmativa. Se seu cão nunca se deparou com a situação e você não é capaz de predizer o comportamento, por favor, use a opção não se aplica.

|  |  | Concordo totalmente | Concordo razoavelmente | Concordo parcialmente/ Discordo parcialmente | Discordo razoavelmente | Discordo totalmente |  | Não se aplica |
| --- | --- | --- | --- | --- | --- | --- | --- | --- |
| 1 | Seu cão raramente fica assustado |  |  |  |  |  |  |  |
| 2 | Seu cão fica bastante excitado quando está prestes a sair para passear (ex. quando vê sua guia, ou quando escuta “passear, etc) |  |  |  |  |  |  |  |
| 3 | Seu cão se assusta facilmente por barulhos e/ou movimentos |  |  |  |  |  |  |  |
| 4 | Seu cão é muito persistente em seus esforços para fazer você brincar com ele |  |  |  |  |  |  |  |
| 5 | Seu cão demonstra pouco interesse em seus arredores |  |  |  |  |  |  |  |
| 6 | Seu cão parece nervoso e/ou sobressaltado por vários minutos depois de ter levado um susto |  |  |  |  |  |  |  |
| 7 | Seu cão se excita facilmente |  |  |  |  |  |  |  |
| 8 | Seu cão tem um medo ou fobia específica |  |  |  |  |  |  |  |
| 9 | Seu cão tenta fugir de casa (jardim) |  |  |  |  |  |  |  |
| 10 | Seu cão parece calmo em lugares barulhentos, cheios |  |  |  |  |  |  |  |
| 11 | Seu cão é cheio de energia |  |  |  |  |  |  |  |
| 12 | Seu cão fica assustado com barulhos de televisão ou radio |  |  |  |  |  |  |  |
| 13 | Seu cão geralmente parece relaxado |  |  |  |  |  |  |  |
| 14 | Seu cão é preguiçoso |  |  |  |  |  |  |  |
| 15 | Seu cão se adapta rapidamente a mudanças em seu ambiente (ex. ser cuidado por uma pessoa diferente, mudar de casa ou ter um membro da família saindo de casa) |  |  |  |  |  |  |  |
| 16 | Seu cão parece ter medo do aspirador de pó ou qualquer outro aparelho doméstico |  |  |  |  |  |  |  |
| 17 | Seu cão necessita de muito encorajamento para participar de atividades energéticas |  |  |  |  |  |  |  |
| 18 | Seu cão persiste em ser travesso apesar de ter levado uma bronca por aquele comportamento |  |  |  |  |  |  |  |
| 19 | Seu cão parece calmo em ambientes não familiares |  |  |  |  |  |  |  |
| 20 | Seu cão é excessivamente animado / brincalhão de maneira descontrolada |  |  |  |  |  |  |  |
| 21 | Seu cão parece inquieto diante de mudanças na sua rotina (ex. se não é alimentado no horário usual, se é deixado sozinho por mais tempo que o usual) |  |  |  |  |  |  |  |
